# Nanocomposites of Fe(II)-Based Metallo-Supramolecular Polymer and a Layered Inorganic–Organic Hybrid for Improved Electrochromic Materials

**DOI:** 10.3390/polym14050915

**Published:** 2022-02-25

**Authors:** Kazuko Fujii, Manas Kumar Bera, Dines Chandra Santra, Masayoshi Higuchi

**Affiliations:** Electronic Functional Macromolecules Group, National Institute for Materials Science, 1-1 Namiki, Tsukuba 305-0044, Ibaraki, Japan; mkb.bera@yahoo.com (M.K.B.); santra.dines@nims.go.jp (D.C.S.)

**Keywords:** metallo-supramolecular polymer, layered inorganic–organic hybrid, phyllosilicate, clay, nanocomposite, electrochromism, optical memory

## Abstract

Fe-based metallo-supramolecular polymer (polyFe), composed of Fe(II) ions and bis(terpyridyl)benzene, is known as a good electrochromic (EC) material. For the first time, to improve the EC properties, we prepared nanocomposites comprising polyFe and a layered inorganic–imidazoline covalently bonded hybrid (LIIm) by simply mixing them in methanol and then examined the effect of the nanocomposition on EC properties. The obtained blue/purple-colored composites (polyFe/LIIm composites) were demonstrated by scanning electron microscopy (SEM) to comprise a structure of LIIm nanoparticles coated with amorphous polyFe. Interestingly, X-ray diffraction (XRD) measurements suggested that there was no intercalation of polyFe in the interlayer space of LIIm. Ultraviolet-visible (UV-vis) spectroscopy measurements demonstrated that light absorption close to 600 nm was attributed to metal-to-ligand charge transfer (MLCT) from the Fe(II) ion to the bisterpyridine ligand and was influenced by LIIm in the composites. The composites exhibited a pair of redox waves, assigned to the redox between Fe(II) and Fe(III), in the cyclic voltammograms; moreover, the composites were estimated to be diffusion controlled. Thin composite films demonstrated reversible EC changes, triggered by the redox reaction of the metal. Furthermore, the results show that the nano-scale composition of the metallo-supramolecular polymers with LIIm can effectively improve the memory properties without reducing the contrast in transmittance (ΔT) of 70–76% in EC changes after applying 1.2 V vs. Ag/Ag^+^. The EC properties varied with varying ratios (3/0.1, 0.5, 1, and 5) of the polyFe/LIIm, and the ratio of 3/1 exhibited the longest memory and largest MLCT absorption peak among composites. The results show that the polyFe/LIIm composites are useful EC materials for dimming glass applications, such as smart windows.

## 1. Introduction

Electrochromic (EC) materials have received considerable attention as an important material used in electrochemical glass tinting, i.e., smart windows and antiglare rearview mirrors in cars [1,2,3,4,5,6,7,8,9,10,11,12,13,14,15,16,17,18,19,20,21,22,23,24,25,26,27,28,29]. Many types of EC materials have been reported and are often categorized as follows. Metal oxides, such as tungsten oxide, are the first generation of EC materials; transition metal complexes, such as Prussian blue, are the second generation of EC materials; and organic molecules and π-conjugated polymers are the third generation of EC materials [3,4,7,8,9,10,11,12]. Recently, metallo-supramolecular polymers (MSPs), composed of metal ions with multi-topic organic ligands, such as bis-terpyridine, were reported as the fourth generation of EC materials [13,14,15,16,17,18,19]. MSPs demonstrate absorption in the visible region because of the metal-to-ligand charge transfer (MLCT) (d → π*) from the metal ion center to the organic ligand. The appearance/disappearance of MLCT absorption is switched by the electrochemical redox of the metal ion in MSPs because the enhanced d–d splitting width is too large to allow the MLCT in the visible region for oxidized MSPs. The colors and redox potential of MSPs are tunable by changing metal ions (such as Fe(II), Ru(II), and Os(II)) or the substituents and spacer of organic ligands [13,14,15,16,17,18,19]. Three-dimensional hyperbranched MSPs show better EC properties than linear MSPs [17]. The high molar extinction coefficient of MLCT absorption in the complex moieties and abovementioned tunability on the bandgap of MLCT have enabled fine EC properties with high optical contrast and abundant color variation. However, the durability and memory properties remain the issues of MSPs, thereby limiting practical applications [20].

**Scheme 1:**
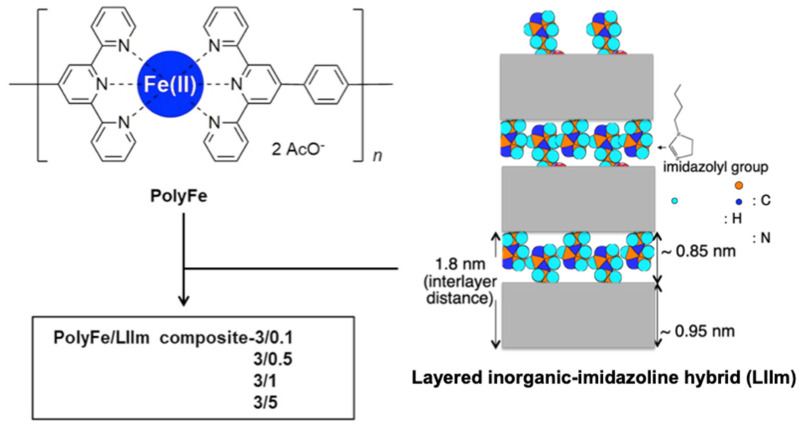
Chemical structures of polyFe and a layered inorganic–imidazoline hybrid (LIIm) in polyFe/LIIm composites.

The formation of composites with inorganic materials is often applied to enhance the physical properties of organic polymer materials [1,21,22,23,24]; however, this method has rarely been utilized for MSPs [25,26,27,28,29]. Previously, we reported a hybrid where an Fe-based metallo-supramolecular polymer (polyFe, Scheme 1) intercalated in the interlayer space of saponite [27,28], a phyllosilicate mineral. For example, Chakraborty et al. reported a polymer nanohybrid material comprising polyFe and tubular hydroxylated halloysite [29]. PolyFe has been incorporated in the hollow lumen and on the external surface of tubular hydroxylated halloysite. Saponite and halloysite are clay minerals, and these hybrids demonstrated electrochromism. Furthermore, combining these clay minerals improved the EC properties. The redox potential (E_1/2_) decreased [27], and optical memory increased [28] via blending with saponite. The combination has achieved a sub-second EC switching and an ultra-high coloration efficiency with the tubular hydroxylated halloysite [29]. However, the optical contrast (ΔT) (for both hybrids) decreased when blending with clay minerals. The decrease can be possibly attributed to polyFe being incorporated in the clay minerals inside (the interlayer space and/or inside of the hollow lumen).

The purpose of this research is to create novel and useful nanocomposites of electrochromic (EC) metallo-supramolecular polymer (MSP) and inorganic materials to achieve superior EC properties, such as large optical contrast (ΔT) or long optical memory. For this purpose, we focused on preparing nanocomposites of MSPs and inorganic materials without intercalation. In this study, we chose a layered inorganic–imidazoline covalently bonded hybrid (LIIm) [30] as the inorganic material. As LIIm has imidazoline moieties covalently immobilized between inorganic layers, it is expected to prevent the undesirable intercalation of MSPs in composites. This study reports the synthesis of polyFe/LIIm composites, the characterization by SEM and XRD, and electrochemical property testing. These features differed from other reported polymer/clay composites [23,29,31]. As for the EC properties, the composites demonstrated redshifting and intensification of MLCT absorption and improved optical memory while maintaining high optical contrast (ΔT).

## 2. Materials and Methods

### 2.1. Materials

PolyFe (Scheme 1) was synthesized as per a previously reported method [14,15] by equimolar complexation between the ditopic ligand 4′, 4‴-(1,4-phenlyene)bis-(2,2′:6′,2″-terpyridine) (Sigma-Aldrich, St. Louis, MO, USA) and iron(II) acetate (Fe(CH_3_COO)_2_, Sigma-Aldrich).

A layered inorganic–imidazoline covalently bonded hybrid (LIIm, Scheme 1) was synthesized as per a previous report [30]. Nickel (II) acetate tetrahydrate (Ni(CH_3_COO)_2_·4H_2_O, >99.9%, FujiFilm-Wako Pure Chemical Industries, Tokyo, Japan) and triethoxy-3-(2-imidazoline-1-yl)propyl silane (C_12_H_26_N_2_O_3_Si, ≥97%, Sigma-Aldrich) reacted at 150 °C for six days. After the reaction, the crude products were washed with purified water several times before being freeze dried. The synthesized LIIm was characterized using inductively coupled plasma optical emission spectrometry (ICP-OES), CHN analysis, thermogravimetric and differential thermal analysis (TG-DTA), X-ray diffraction (XRD), scanning electron microscopy (SEM), Fourier transform infrared (FT-IR) spectroscopy, ultraviolet-visible-near-infrared (UV-VIS-NIR) spectroscopy, and fluorescence spectrophotometry, which are all described in detail in [30]. These results have led to a proposed model for LIIm, which is a layered structure comprising inorganic layers with an imidazolyl group sandwiched between them, with an interlayer distance of 1.8 nm. The imidazolyl group is anchored to the layer surface of the inorganic layer via a Si–C covalent bond. The inorganic layer comprises a Ni^2+^ octahedral sheet sandwiched between two siloxane sheets.

Methyl alcohol (MeOH, >99.8%) and spectroscopy-grade acetonitrile (ACN) (>99.8%) were purchased from FujiFilm-Wako Pure Chemical Industries. Battery grade lithium perchlorate (LiClO_4_, >99.0%)) was purchased from FujiFilm-Wako Pure Chemical Industries. Indium tin oxide (ITO)-coated glasses (sheet resistance: 8–12 Ω/cm^2^) were purchased from Sigma-Aldrich.

### 2.2. Preparation of PolyFe/LIIm Composite Films

An amount of 30 mg of polyFe was dissolved in 10 mL of MeOH via stirring for 3 h at room temperature (RT) to obtain the stock solution of polyFe with a concentration of 3 mg/mL. Separately, four stock dispersions of LIIm were prepared by dispersing LIIm in MeOH with ratios of LIIm/MeOH of 0.1, 0.5, 1, and 5 mg/mL. An amount of 500 μL of the polyFe stock solution with a concentration of 3 mg/mL was mixed with 500 μL of the stock dispersion of LIIm in MeOH with ratio of 0.1 mg/mL. The mixtures were stirred at RT for 24 h and then cast and spin coated on ITO glass (cast and spin coating) and quartz glass (cast) to obtain polyFe/LIIm composite-3/0.1 films (Scheme 1). Spin coating was performed at a coating speed of 120 rpm for 900 s with 160 μL of the mixture after stirring. Other polyFe/LIIm composite films (3/0.5, 1, and 5 ratios) were obtained by the same procedure using stock dispersions of LIIm with 0.5, 1, and 5 mg/mL ratios.

Reference films were prepared on ITO glass and quartz glass with a MeOH solution of polyFe using the same procedure, except that 80 μL of the MeOH solution of polyFe was used for spin coating.

The cast films on ITO glass were used for obtaining cyclic voltammogram (CV), and the spin-coated films were used for EC characterizations. The cast films on quartz glass were used for ultraviolet-visible (UV-vis) spectroscopy and XRD.

### 2.3. Characterization

The SEM images of the obtained polyFe/LIIm composites and pure polyFe (for reference) drop casted on a Si wafer and a powder sample of LIIm only (for reference) on carbon tape were obtained using HITACHI S-5000, HITACHI FE-SEM S-4800, and SU-8000 at accelerating voltages of 10 and 5 kV. Before SEM observation, specimens were coated with a thin layer of Pt. XRD measurements were conducted for the cast films on quartz glass at room temperature under a dry nitrogen flow using CuKα radiation (λ = 1.5405 Å) on a Rigaku RINT-2200 HF X-ray diffractometer equipped with a humidity- and temperature-controlled chamber [32].

UV-vis spectra were obtained for the cast films on the quartz glass using a Jasco V-670 UV/VIS/NIR spectrophotometer. An integrating sphere was used for reflectance measurements.

CV was performed on an ALS/CHI electrochemical workstation (CH Instruments, Inc., Austin, TX, USA) to examine the redox properties of the polyFe/LIIm composites. A conventional three-electrode system was set with the polyFe/LIIm composite cast films on ITO glass as working electrodes, spring-type Pt wire as the counter electrode, and an Ag/Ag^+^ electrode saturated with acetonitrile, including 0.1 M tetrabutylammonium perchlorate (TBAP) + 0.01 M AgNO_3_, as the reference electrode. The electrolyte solution was 0.1 M LiClO_4_ in anhydrous acetonitrile. Scan rates were set at 5, 10, 50, 100, and 500 mV/s and 1 V/s.

A VersaSTAT4 (AMETEK Inc., Berwyn, PA, USA) was used with an Ocean Optics modular spectrometer to record EC properties, e.g., optical changes on potential alteration. A conventional three-electrode system was set in a manner similar to the CV measurement, except for the use of the spin-coated composite films (the active areas were 1.5 × 1.5 cm^2^) as a working electrode. Transmittance was observed on applying a potential of 0 and 1.2 V and after applying the potential.

## 3. Results and Discussion

### 3.1. Structural Characterization

**Figure 1:**
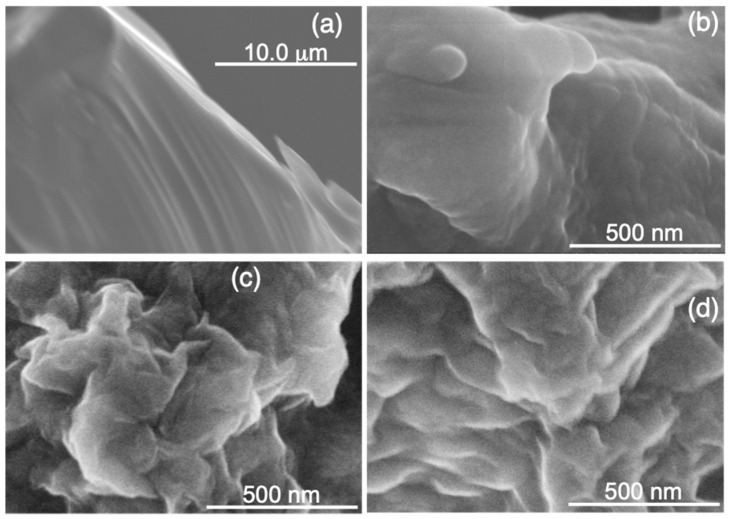
SEM images of polyFe/LIIm composites (**a**) 3/0.1, (**b**) 3/0.5, (**c**) 3/1, and (**d**) 3/5.

To discuss the relationship between the complexed structure and EC properties of nanocomposites, we first performed SEM and XRD. The SEM images revealed different morphologies and particle sizes among the polyFe/LIIm composites (Figure 1). Large particles with sizes of 15–30 μm (an average size of 24 μm) were observed for the polyFe/LIIm composite-3/0.1 (Figure 1a). A large portion of polyFe was spread on the Si wafer used for SEM observations (Figure S1a, Supplementary Information (SI)). Agglomerates were partially observed for polyFe only (Figure S1b, SI). The sizes of pure polyFe agglomerates were 3–175 μm (an average size of 40 μm). The surface was bumps and dips for the pure polyFe agglomerates (Figure S1b, SI). The SEM image for the polyFe/LIIm composite-3/0.1 (Figure 1a) demonstrated a relatively angular shape and smoother surface with crimps-like draped curtain and without the bumps and dips compared with the pure polyFe agglomerates (Figure S1b, SI). The differences in morphologies between polyFe/LIIm composite-3/0.1 and pure polyFe could be attributed to combining polyFe with LIIm, although LIIm was rarely observed for the polyFe/LIIm composite-3/0.1.

The SEM images of LIIm demonstrated layered morphologies with a size range of 60–300 μm and with an average particle size of 130 μm that comprised stacking lamella with sizes of 26–140 μm (Figure S2a, SI). An SEM image for LIIm with higher magnification (Figure S2b, SI) demonstrated smaller planular particles with an average size of 290 nm (120–830 nm) stacked in the stacking direction and without order in the lateral direction to form layered morphologies.

The surface seems to be smoother for polyFe/LIIm composite-3/0.1 (Figure 1a) than that for LIIm only (Figure S2a,b, SI). The SEM images of LIIm only (Figure S2a,b, SI) demonstrated a rough surface because of the stacking of LIIm smaller planular particles.

For the polyFe/LIIm composite-3/0.5, the SEM images revealed aggregations, such as sliding layered aggregates sized at several micrometers (Figure 1b). An SEM image of the polyFe/LIIm composite-3/0.5 with a higher magnification (Figure S3a, SI) demonstrated that the smaller planular particles with an average size of 342 nm (from 252 to 420 nm) were stacked in the stacking direction and without order in a lateral direction to form the sliding layered morphologies. Each of the smaller planular particles in the polyFe/LIIm composite-3/0.5 demonstrated slightly rounded edges and a smoother surface compared with those of the LIIm only (Figure S2b, SI).

PolyFe/LIIm composites-3/1 and 3/5 demonstrated disordered agglomerates (Figure 1c,d). The polyFe/LIIm composite-3/5 had larger agglomerates with sizes of 1–13 μm (an average size of 5.7μm) than polyFe/LIIm composites-3/0.5 and 3/1 with sizes of 1–8 μm (an average size of 3 μm). The images of agglomerates for polyFe/LIIm composite-3/1 (Figure 1c) were more disordered than the polyFe/LIIm composites-3/0.5 and 3/5 (Figure 1b,d). For the polyFe/LIIm composite-3/1, the disordered agglomerates comprised smaller planular particles with an average size of 240 (from 70–570) nm (Figure S4, SI). The SEM image (Figure 1d and Figure S3b, SI) shows that the large agglomerates comprised layered aggregates with sizes of 0.5–5 μm (an average size of 1 μm), and the layered aggregates comprised smaller planular particles with an average size of 270 (80–560) nm for polyFe/LIIm composite-3/5. Because the agglomerates were disordered for the polyFe/LIIm composites-3/1 and 5, the thicknesses of the smaller planular particles were relatively easily observed. The average thicknesses were 24 (6–58) and 18 (8–36) nm for polyFe/LIIm composites-3/1 and 5, respectively. The SEM images of polyFe/LIIm composite-3/1 and 5 (Figure 1c,d and Figure S3b, SI) demonstrated a smoother surface and slightly rounded edges compared with those of the LIIm only (Figure S2, SI).

SEM observations demonstrated well-ordered larger layered aggregations with an average size of 1.8 μm (0.8–3 μm) and large particles with morphologies similar to those seen in polyFe/LIIm-3/0.1 (Figure 1a), but with smaller sizes of ~5 μm for the polyFe/LIIm composite-3/5 (not shown). Thus, SEM studies indicated the polyFe/LIIm-3/5 was inhomogeneous.

SEM observations demonstrated that the morphologies of polyFe/LIIm composites differed from both pure polyFe and LIIm. The morphologies varied with polyFe/LIIm ratios. The polyFe/LIIm composite-3/1 exhibited the most disordered agglomerates among all composites. The surface was smoother, and the edges were slightly rounded for the polyFe/LIIm composites compared to those of the LIIm only. The surfaces of the polyFe/LIIm composites and LIIm alone would have different compositions and structures.

**Figure 2:**
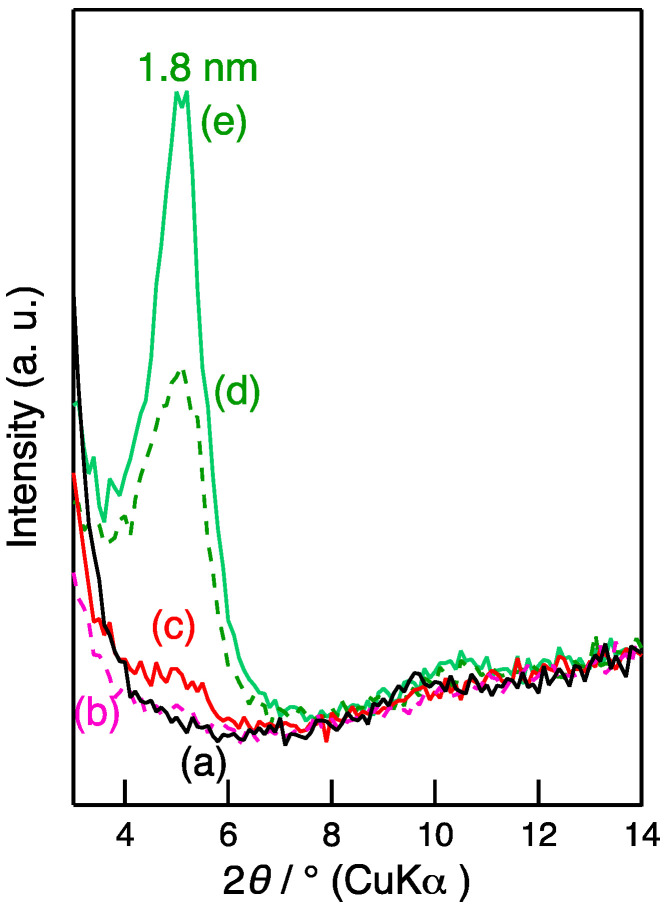
XRD patterns of polyFe/LIIm composites (**a**) 3/0.1, (**b**) 3/0.5, (**c**) 3/1, and (**d**) 3/5 and (**e**) a reference LIIm cast films on quartz glass.

The XRD peak was observed at a low angle (d = 1.8 nm) for LIIm (Figure 2e) and is attributed to the interlayer distance, including the layer thickness and gallery height between the inorganic moieties of LIIm (Scheme 1) [30]. The XRD peak was not observed for the polyFe/LIIm composite-3/0.1 (Figure 2a) and barely observed for the polyFe/LIIm composite-3/0.5 (Figure 2b). The XRD peak was observed for the polyFe/LIIm composites-3/1 and 5. The XRD peak was not shifted for the composites compared with LIIm only. The gallery height (Δd) between the inorganic moieties of LIIm can be estimated at 0.85 nm by assuming that the thickness of the inorganic moiety is the same as the 0.95 nm [33] thickness of the 2:1 layer smectites. The theoretical thickness of polyFe was calculated using MM3 as 0.9 nm [27]. It is difficult for the polyFe to be intercalated in the interlayer with a distance of 1.8 nm. The XRD peak weakened as the ratios of LIIm to polyFe decreased. The XRD peak intensity decreased more significantly than expected based on the reduction in the ratios of LIIm in polyFe/LIIm composites. Two reasons are proposed for the reduction in the intensity of the XRD peak with decreasing ratios of LIIm to polyFe in the composites: (1) the contents of LIIm were extremely low in the composites to indicate the XRD peak, and (2) there were no layered aggregates in the composites with lower LIIm contents (polyFe/LIIm composites-3/0.1 and 0.5). Stronger scatterings were observed in the lowest angle region for polyFe/LIIm composites-3/0.1, 3/1, and 3/0.5.

**Scheme 2:**
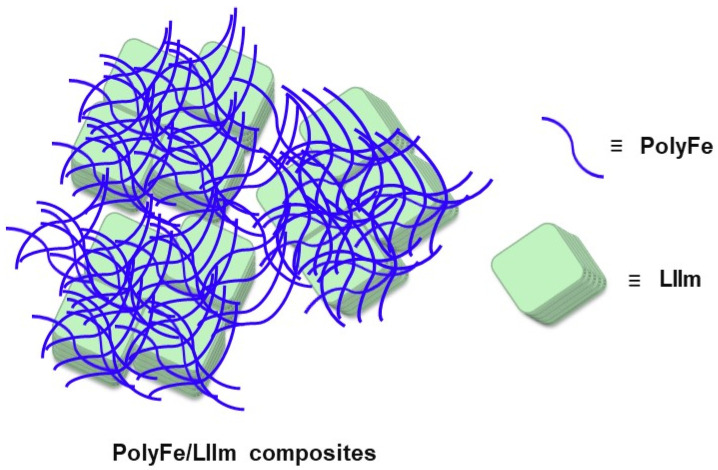
A schematic of polyFe/LIIm composites.

These results show that most polyFe is not intercalated in the interlayer space. SEM observations demonstrated a smoother surface and slightly rounded edges for polyFe/LIIm composites compared to those of LIIm only (Figure 1). Based on these experimental results, one can speculate that amorphous polyFe coats the smaller planular particles, layered aggregates, and disordered agglomerates of LIIm in polyFe/LIIm composites (Scheme 2). Because of this coating, it was more difficult to recognize the layered aggregates and smaller planular particles of LIIm with increasing ratios of polyFe to LIIm.

The observed XRD peaks with a d value of 1.8 nm (Figure 2c,d) show that LIIm layers were stacked in an order in the stacking direction in the smaller planular particles in polyFe/LIIm composites-3/1 and 5 at least in the major part. As described above, the disordered agglomerates and layered aggregates comprised smaller planular particles with average sizes of 240 and 270 nm and average thicknesses of 24 (6–58) and 18 (8–36) nm for polyFe/LIIm composites-3/1 and 5 (Figure 1c,d). One smaller planular particle comprised stacking 14 (4–33) and 11 (5–21) LIIm layers on average (ranges) for polyFe/LIIm composites-3/1 and 5, respectively, based on the average thicknesses and an interlayer distance of 1.8 nm (Figure 2). It is possible that higher scattering may be attributed to the partially exfoliated LIIm.

As per SEM images, the agglomerates of polyFe/LIIm composites-3/1 and 5 were larger and more disordered than aggregates of polyFe/LIIm composite-3/0.5. These variable morphologies are related to differences between the preparation media with different concentrations, as discussed below. For preparing polyFe/LIIm composites-3/0.5, 3/1, and 3/5, the LIIm dispersions in MeOH were not transparent. With MeOH, LIIm is minimally swollen and exfoliated. Accordingly, we may assume that most of LIIm’s smaller planular particles comprising stacking LIIm layers will not be exfoliated in the preparation media. In the preparation medium with a dilute concentration of LIIm for polyFe/LIIm composite-3/0.5, the dispersed smaller planular particles would be covered by polyFe and then simultaneously gather to form the reasonably ordered layered aggregates. Because the content of LIIm (smaller planular particles and aggregates) in the preparation medium was high, the agglomerates for polyFe/LIIm composites-3/1 and 3/5 were larger and more disordered than the polyFe/LIIm composite-3/0.5. Because the content of the polyFe/LIIm composite-3/5 preparation medium was high, in the beginning, the layered aggregates of LIIm did not entirely collapse into smaller planular particles. Therefore, until the stirring was completed in the preparation operation, the layered aggregates still existed. In the preparation technique for polyFe/LIIm composite-3/5, the layered aggregates of LIIm would congregate in huge agglomerates, resulting in more organized morphologies in SEM images than in the polyFe-LIIm composite-3/1.

Although many composites comprising polymers and inorganic materials have been reported, there have been few reports of composites of polymers coating inorganic materials without a special process. Inorganic particles were dispersed in the polymer matrices in many of the reported composites [31]. We consider polyFe could coat the smaller planular particles, aggregates, and agglomerates of the negatively charged LIIm to combine in polyFe/LIIm composites with such an interesting feature because polyFe is positively charged.

In our previous study, polyFe was intercalated in the interlayer space of saponite to generate a saponite-polyFe composite [27]. While preparing the saponite-polyFe composite, saponite is dispersed in water. In water, saponite can swell and exfoliate. For this study, LIIm was diffused in MeOH. Because the imidazolyl group lies in the interlayer gap and is anchored to the layer of LIIm, it rarely swells and exfoliates in MeOH. Consequently, polyFe is rarely intercalated in the interlayer region of LIIm, resulting in polyFe/LIIm composites with an intriguing phenomenon that differs from clay-polyFe composites previously reported. The possibility of exfoliation will be discussed in the section below.

As reported in this subsection, SEM observations and XRD measurements demonstrated that polyFe coated the outside of the smaller planular particles, layered aggregates, and disordered agglomerates of LIIm to form polyFe/LIIm composites. The morphologies were varied for each polyFe/LIIm composite with varying polyFe/LIIm ratios. The morphologies of the polyFe/LIIm composite-3/0.1 were completely different compared to the other three composites. SEM studies demonstrated the polyFe/LIIm composite-3/5 was inhomogeneous. The polyFe/LIIm composite-3/1 exhibited the most disordered agglomerates among these composites.

### 3.2. UV-Vis Spectroscopy

**Figure 3:**
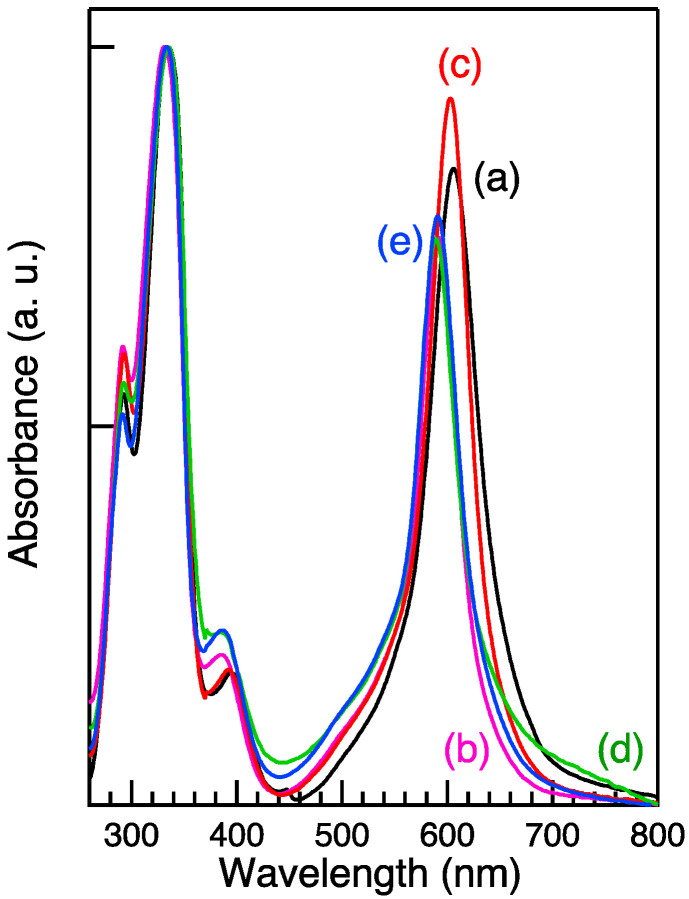
UV-vis spectra for polyFe/LIIm composites (**a**) 3/0.1, (**b**) 3/0.5, (**c**) 3/1, and (**d**) 3/5 and (**e**) reference polyFe films cast on quartz glass. The vertical axis is normalized with absorbance at ~335 nm.

Absorption peaks appeared in the UV-vis region for polyFe/LIIm composites and polyFe only (Figure 3). The UV-vis spectra were normalized with the absorbance of peaks close to 335 nm in Figure 3. The absorption peaks at ~335 nm are attributed to the π–π* transition of the ligand of the polyFe part. The UV-vis spectra showed that the absorption at ~600 nm was attributed to MLCT (d → π*) from Fe(II) ion to terpyridine moiety for polyFe/LIIm composites and polyFe. Interestingly, the MLCT absorptions (the intensities of normalized absorbance) were enhanced by combining with LIIm about polyFe/LIIm composites-3/1 and 3/0.1 (Figure 3a,c). The absorbance of the MLCT absorption was comparable with that of the π–π* absorption for polyFe/LIIm composites-3/1, although the MLCT absorptions are significantly weaker than π–π* absorption in many cases for Fe-based complexes [17] and polyFe–clay hybrids [27]. The absorption close to 385 nm is attributed to the metal (Fe(II)) d–d transition of the polyFe part [15,27].

The absorption peaks of MLCT bands and d–d transition were redshifted for the polyFe/LIIm composites-3/0.1 (15 nm) and 3/1 (12 nm). These results suggest that the band gap energy decreased for the polyFe/LIIm composites-3/0.1 and 3/1. The absorptions attributed to the MLCT band were stronger, and absorptions caused by the d–d transition were weaker for the polyFe/LIIm composites-3/0.1 and 3/1 compared with polyFe only and other polyFe/LIIm composites-3/0.5 and 3/5. These changes suggest that transition probabilities are higher for the MLCT band and lower for the d–d transition about the polyFe/LIIm composites-3/0.1 and 3/1.

Different counter anions exert different influences on the bandgap energies of polyFe. Changes in these complexes, e.g., distortion in the octahedral structure, influence the bandgap energies [13]. Redshifts for dyes and complexes immobilized on solid [34] and dense solutions have been reported, and it has been demonstrated the redshifts are related to changes in their structures and the restriction of their mobilities, e.g., rotation and stabilization by the surrounding solids, such as solvation. We could speculate that the redshift of the MLCT band could be attributed to the exchange of the counter anion from the acetate anion to LIIm and, therefore, by stabilizing polyFe with LIIm. The absorption peaks attributed to the π–π* transition were not shifted. The results should demonstrate that the energy levels should not change for both the π and π* orbitals. When the energy level is stabilized for the d orbital but does not change for the π* orbital, the absorption should blueshift about the MLCT. Currently, the reason for the redshift in the MLCT absorption is being debated.

Interestingly the MLCT absorptions were enhanced and redshifted for polyFe/LIIm composites-3/1 and 0.1. The trend did not depend on the increasing amount of LIIm in composites. The complex trend could be related to the characteristic combining features with the most disordered morphologies for polyFe/LIIm composite-3/1. We will discuss this in detail below (Section 3.5). We should note that the absorption of Ni(II) of LIIm should be overlapped on the absorption at ~385 nm. The broad absorption peaks appear at ~390 nm and 685 nm and are attributed to d–d transitions ^3^A_2g_ → ^3^T_1g_ (P) and ^3^A_2g_ → ^3^T_1g_ (F) of Ni(II) for LIIm [30]. The UV-vis spectrum demonstrated a tailing for the MLCT band of the polyFe/LIIm composite-3/5 in a longer wavelength side (Figure 3d). The tailing should be attributed to the overlap of absorption of the d–d transition ^3^A_2g_ → ^3^T_1g_ (F) of Ni(II). The absorption close to 385 nm is broad for the polyFe/LIIm composite-3/5. The broadening would have been attributed to the overlap with the broad absorption of ^3^A_2g_ → ^3^T_1g_ (P) of Ni(II). The overlap should have had more of an effect as the contents of LIIm increased in the polyFe/LIIm composites. Therefore, we recognized the overlaps for the polyFe/LIIm composite-3/5. Reflection absorption spectra were measured for the same samples. The overlaps were recognized for polyFe/LIIm composite-3/5 in the reflection absorption spectra (not shown).

PolyFe/LIIm composites exhibited a MLCT band at ~600 nm. Interestingly, the MLCT absorptions were enhanced and redshifted for polyFe/LIIm composites-3/1 and 3/0.1.

### 3.3. CV

**Figure 4:**
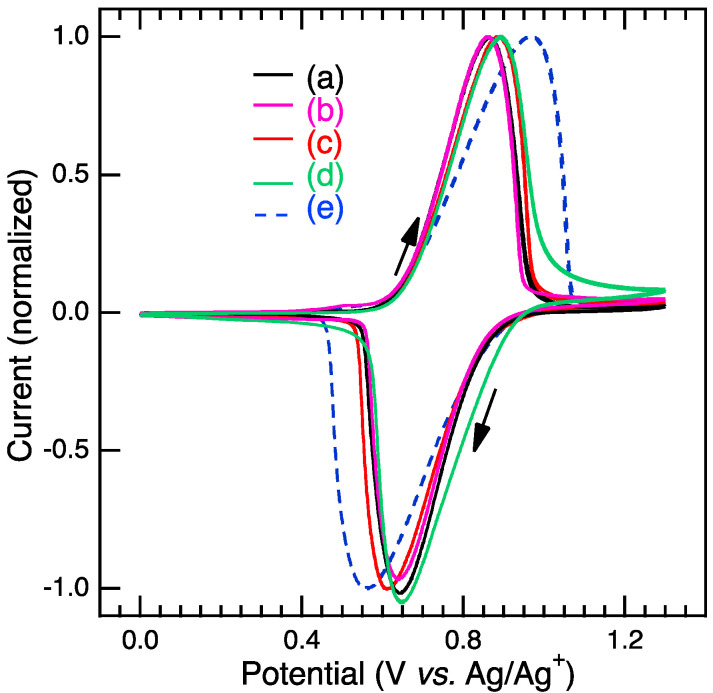
CV curves of polyFe/LIIm composites (**a**) 3/0.1, (**b**) 3/0.5, (**c**) 3/1, and (**d**) 3/5 and (**e**) reference polyFe films cast on ITO glass in a three-electrode system with a spring-type Pt wire as a counter electrode and Ag/Ag^+^ as a reference electrode in 0.1 M LiClO_4_ of anhydrous acetonitrile solution as an electrolyte solution with a scan rate of 50 mV/s. Peak currents within the vertical axis normalize these CV curves.

In the cyclic voltammograms, a pair of redox peaks appeared at 0.86–0.89 V vs. Ag/Ag^+^ for polyFe/LIIm composite cast films and 0.96 V for the reference polyFe cast film when applying potential to the positive side (scan rate: 50 mV/s) (Figure 4). Peak currents in the vertical axis normalized the CV curves. The polyFe/LIIm composite and reference polyFe cast films turned transparent from blue-purple color around the peak when applying potential. The color change is attributed to the oxidation of Fe(II) ion to Fe(III) ion [14,15]. The peak potentials in CV are described to be oxidation potentials.

Color changes occurred at lower potentials for polyFe/LIIm composite cast films than the reference polyFe cast film. When the potential was applied to the opposite side (from 1.3 to 0 V), peaks appeared (Figure 4), along with a color change to blue-purple from transparent. The changes in current and color were reversible, although Figure 4 shows only the second cycle. Thus, polyFe/LIIm composite cast films, as well as polyFe, demonstrated reversible electrochromism.

CV measurements did not show any drastic change in half-wave potentials (E_1/2_) for polyFe/LIIm composite cast films compared with the reference polyFe cast film. E_1/2_ was 0.75–0.77 V for polyFe/LIIm composite cast films and 0.76 V for the reference polyFe cast film. The oxidation potential decreased, and the redox potential increased for polyFe/LIIm composite cast films compared with the reference polyFe cast film. Gaps between the oxidation and reduction potentials (ΔE) considerably decreased. ΔEs were 0.22–0.27 V for polyFe/LIIm composite cast films and 0.40 V for the reference polyFe cast film. Based on the reduction in ΔE, one can consider electron transfer at the interface between films and electrode becomes faster in the composites. ΔEs slightly increased for the previously reported polyFe–clay composites [27,29].

**Figure 5:**
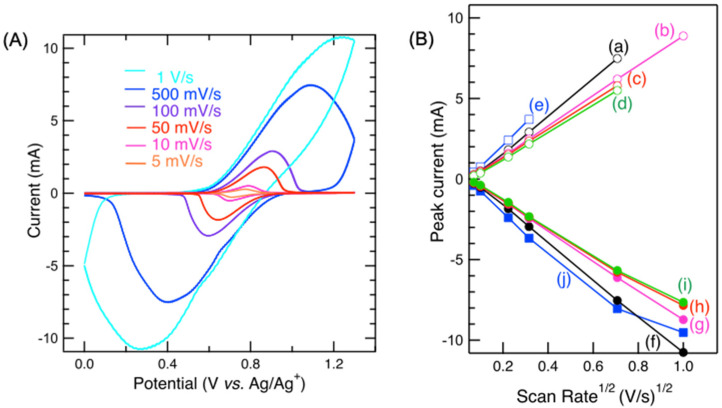
(**A**) CVs of polyFe/LIIm composite-3/0.1 film casted on ITO glass in the three-electrode system with diverse scan rates of 5, 10, 50, 100, and 500 mV/s and 1 V/s. (**B**) CV plots of the oxidation (**a**–**e**) and reduction (**f**–**j**) current as a function of the square root of the scan rates for polyFe/LIIm composites (**a**,**f**) 3/0.1, (**b**,**g**) 3/0.5, (**c**,**h**) 3/1, and (**d**,**i**) 3/5 and (**e**,**j**) reference polyFe films casted on ITO glass.

Scan-rate-dependent CV measurements were performed to better understand the redox mechanism (Figure 5). The oxidation and reduction potentials shifted more as the scan speeds increased, and the peak currents increased for both polyFe/LIIm composite cast films and polyFe cast films (Figure 5A). Both anodic and cathodic peak currents were linearly related to the square root of scan rates (Figure 5B), and the scan rates were not proportional to the anodic and cathodic peak currents (Figure S5, SI). Diffusion-controlled redox systems are shown by the linear dependency of peak currents on the square root of scan rates. The rate-determining step can be represented as the diffusion of counter anion(s).

With increase in the concentration of LIIm, the peak currents decreased to 0.75 (polyFe/LIIm composite-3/0.1)–0.57 (polyFe/LIIm composite-3/5) times that of polyFe, and slopes of the plots of peak currents vs. the square root of the scan rates decreased (Figure 5B). It has been concluded based on Fick’s second law and the Nernst equation that the slope is proportional to diffusion coefficient in the diffusion-controlled system. The smaller slopes demonstrate that the counter anion(s) diffusion was slower by combining polyFe with LIIm. The slower diffusion of the counter anion (the acetate ion) would be primarily attributed to the presence of LIIm in the composites. We speculate the positive charge of polyFe could be compensated with the inorganic layer moiety with the negative charge in part and the acetate ion in polyFe/LIIm composites. Here, the counter anion (LIIm) would hardly diffuse.

The currents in the CV curves decreased but did not drastically decrease for the polyFe/LIIm composites compared with pure polyFe. Drastic decreases (~0.2 times) in the currents for polyFe intercalated in the interlayer space of saponite [27] have been reported. It has been discussed in the literature [27] that electron hopping rate could decrease by the layer space and by the less mobile counter anion, which should exist between layers in the SP–polyFe hybrid. The SEM and XRD experimental results demonstrated polyFe coats the LIIm aggregates in polyFe/LIIm composites. This would be a reason why the currents did not drastically decrease.

To conclude this subsection, polyFe/LIIm composites demonstrated a reversible pair of redox waves with accompanying reversible color changes from blue-purple color and transparent upon the application of potential with E_1/2_s of 0.75–0.77 V, which were comparable to that of polyFe, and ΔEs of 0.22–0.27 V, which were significantly smaller than that of polyFe. The reactions for electrochromism were diffusion-controlled redox systems with slower counter anion diffusion in polyFe/LIIm composites.

### 3.4. EC Properties

**Figure 6:**
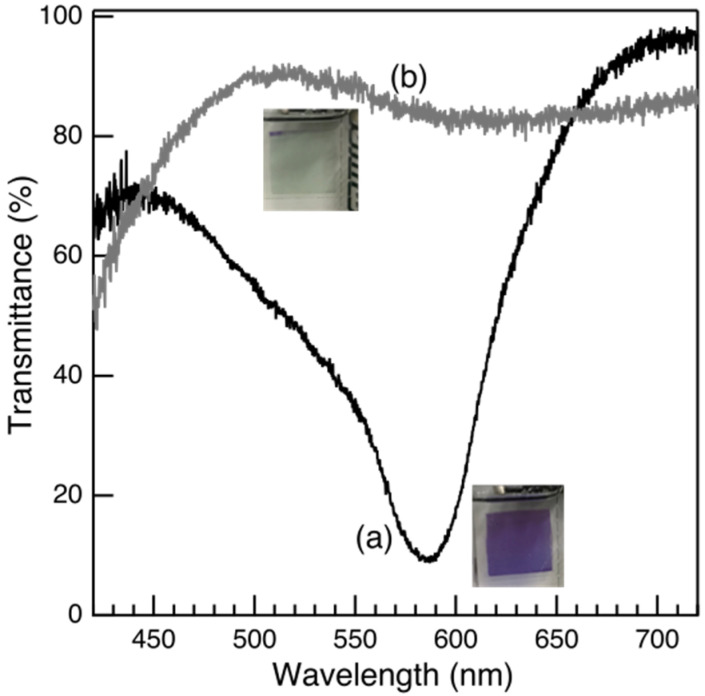
Transmittance spectra recorded on an Ocean Optics modular spectrometer for polyFe/LIIm composite-3/0.1 film spin coated on ITO glass (1.5 × 1.5 cm) (**a**) before (color state) and (**b**) after oxidation (bleached state) upon applying 1.2 V vs. Ag/Ag^+^ by chronoamperometry on VersaSTAT4 in the three-electrode system with a spring-type Pt wire as a counter electrode and Ag/Ag^+^ as a reference electrode in 0.1 M LiClO_4_ of anhydrous acetonitrile solution as an electrolyte solution. Inset: the photographs.

The blue-purple polyFe/LIIm composite-3/0.1 spin-coating film turned transparent upon applying a potential of 1.2 V vs. Ag/Ag^+^ (insets of Figure 6). Simultaneously, in situ spectro-electrochemical measurement demonstrated transmittance at 587 nm and became higher at 84.7% upon the applied potential of 1.2 V vs. Ag/Ag^+^ from 8.7% before the applied potential (Figure 6a,b). MLCT absorption disappeared upon the applying potential. The blue-purple color and MLCT absorption reappeared upon the applied potential being decreased to 0 V vs. Ag/Ag^+^. Similarly, other polyFe/LIIm composites-3/0.5 and 1 spin-coating films demonstrated color changes and disappearance of MLCT absorption when potentials were applied. Another polyFe/LIIm composite-3/5 spin-coating film demonstrated a color change, but the polyFe/LIIm composite-3/5 spin-coating film was inhomogeneous upon applying the potential. There were blue-purple spots in the polyFe/LIIm composite-3/5 spin-coating film even upon using the potential.

For polyFe/LIIm composite-3/0.1, 0.5, and 1 spin-coating films, the transmittance contrasts (ΔT) between colored and bleached states were 76%, 70%, and 74%, respectively. The polyFe spin-coating film had a ΔT of 74%, and the ΔTs of polyFe/LIIm composites were comparable with polyFe film. They have previously reported that the polymer nanohybrids of polyFe have reduced ΔT. PolyFe was incorporated within a tube and on an outer surface of tubular hydroxylated halloysite [29]. PolyFe covered the aggregation and agglomerations of LIIm in polyFe/LIIm composites. Therefore, large optical contrasts (ΔT) were maintained for polyFe/LIIm composites. Hu et al. previously reported hierarchically porous electrochromic film of nanocomposites comprising poly(3,4-ehylendioxythiophene) (PEDOT) coating on outer halloysites nanotubes with significantly higher ΔT of 59.3% than that of 15.3% for pure PEDOT [23]. In this study, because we employed polyFe with a considerably high ΔT of 72% [29] and polyFe coated the outer side of the aggregations and agglomerates of LIIm, polyFe/LIIm composites with high ΔT were achieved.

**Figure 7:**
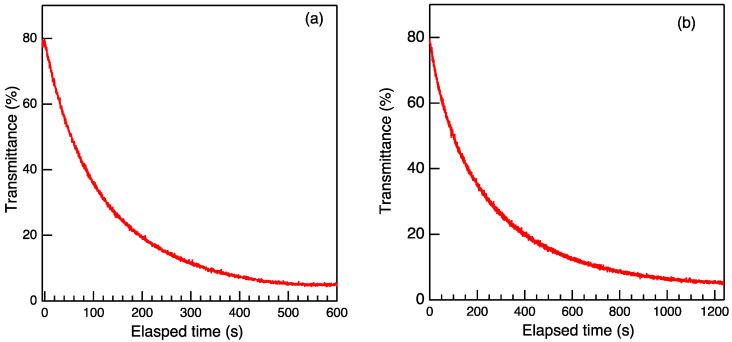
Transmittance decay curves after switching off the applied voltage at 1.2 V vs. Ag/Ag^+^ at the bleached (oxidized) to colored states at (**a**) closed- and (**b**) open-circuit conditions of polyFe/LIIm composite-3/1 film spin coated on ITO glass (1.5 × 1.5 cm) in the three-electrode system with a spring-type Pt wire as a counter electrode and Ag/Ag^+^ as a reference electrode in 0.1 M LiClO_4_ of anhydrous acetonitrile solution as an electrolyte solution.

PolyFe/LIIm composites demonstrated significantly longer EC memory (Figure 7). It took 785 s for polyFe/LIIm composite-3/1 film to recover 95% of the colored state in the open-circuit condition, although it took 236 s for the polyFe film. The logarithm of the transmittance (log T) exhibited linear decay vs. the elapsed time, i.e., the concentration of Fe^2+^ ion linearly increased vs. time after oxidation (to re-reduction).

The retention times of the EC memory changed with polyFe/LIIm ratios of polyFe/LIIm composites, as listed in Table 1. PolyFe/LIIm composites-3/0.1, 1, and 5 exhibited longer EC memories than polyFe in open and closed circuits. However, polyFe/LIIm composite-3/0.5 exhibited shorter EC memory than polyFe. The decays in the Fe(III) center were slower for the polyFe/LIIm composite-3/0.1 (closed-circuit), 3/1, and 3/5 films (both circuits) than the polyFe film. To reduce Fe^3+^ centers, electron transfer on the interface between the films and electrodes is necessary, as is the diffusion of the counter anion(s) [18]. The smaller ΔEs suggest that the electron transfers from the electrode to films were faster for polyFe/LIIm composite films than polyFe film. The smaller slopes (Figure 5B) indicated the slower diffusion of counter anion(s) for polyFe/LIIm composite films. The shorter EC memory of polyFe/LIIm composite-3/0.5 would be attributed to the faster electron transfer on the electrode/composite film interface. This effect would influence the reduction rate more strongly than another effect by the slower diffusion of counter anion(s). The longer EC memories of other polyFe/LIIm composite films would be attributed to the slower diffusion of counter anion(s). Thus, the longer EC memories were achieved without the decrease in ΔT (larger than and/or equal to 70%) by combining polyFe with LIIm. However, retention times did not simply depend on the contents of LIIm. We will discuss this below.

### 3.5. The Improved Properties and Combining Features

The combination of polyFe with LIIm influenced the properties, i.e., the intensified and redshifted MLCT absorption, redshifted and weakened d–d transition absorption, and longer electrochromism memory while maintaining a large optical contrast (ΔT). However, the influence did not gradually enhance with increase in the LIIm contents. The stronger effects were detected for polyFe/LIIm composites-3/1 and 3/0.1.

The structural features were different for each polyFe/LIIm composite but did not gradually change with the LIIm contents. The experimental results suggested the particles of LIIm would be smaller in polyFe/LIIm composites-3/0.1 and 3/1 compared with polyFe/LIIm composites-3/0.5 and 3/5. The XRD measurements showed stronger scattering in the lowest angle region for polyFe/LIIm composites-3/0.1 and 3/1 (Figure 2). The SEM observation demonstrated more disordered agglomerates for polyFe/LIIm composite-3/1 than polyFe/LIIm composites-3/0.5 and 3/5 (Figure 1). The disordered agglomerates comprised smaller planular particles for polyFe/LIIm composite-3/1. The disordered agglomerates comprised layered aggregates and smaller planular particles for polyFe/LIIm composites-3/5 and 3/0.5. The surface area of LIIm contacting polyFe increased in the composites, when the smaller planular particles of LIIm were more dominant than the layered aggregates. We speculate that these differences in the combining features would be a reason for the stronger influence on polyFe/LIIm composites-3/1 and 3/0.1.

The larger effects in polyFe/LIIm composite-3/0.1 may evoke one possibility, where LIIm may be intercalated and exfoliated by polyFe in part. Indeed, this is consistent with the XRD data with relatively high scattering in the lowest angle region for polyFe/LIIm composites-3/0.1 and 3/1. LIIm is hardly swelled and exfoliated in MeOH; however, we speculate that LIIm would be possibly swelled and partly exfoliated in the mixture with a countless amount of MeOH and polyFe. We speculate that affinities between MeOH and Si–OH groups on the layer surface of LIIm and between the polyFe and imidazolyl group of LIIm could act as driving forces for swelling and exfoliation. There is an Si–OH group in the proposed structure for LIIm because of the steric hindrance of the imidazolyl group [30], although there is no Si–OH group on the layer surface in the ideal structure of 2:1 phyllosilicates [33,34,35,36]. LIIm, partially intercalated and exfoliated, would strongly influence the MLCT and d–d transition bands, e.g., stabilizing the orbitals compared with LIIm comprising only the larger particles. LIIm would act as a counter anion of polyFe and stabilize the oxidized Fe(III) state, such as the electron-donating groups [13]. The EC memory would be longer by stabilizing the oxidized Fe(III) state.

The EC memory properties were improved (Figure 7) by combining with insulated LIIm. We consider the slower diffusion (Figure 5B) of counter anion(s) in polyFe/LIIm composite films as the primary reason for improvement. Stabilizing the oxidized Fe(III) state by LIIm could be an additional reason for the long memory. When LIIm was the smaller planular particles and partly intercalated and exfoliated by polyFe, polyFe would be influenced more strongly by LIIm and the oxidized Fe(III) state was more stabilized.

The stronger combining effects on the properties were demonstrated for polyFe/LIIm composites-3/1 and 3/0.1 rather than effects gradually depending on the increasing ratios of LIIm. The combined features with smaller particles and higher surface area of LIIm contacting polyFe in polyFe/LIIm composites-3/1 and 3/0.1 would exhibit stronger effects.

## 4. Conclusions

We prepared novel nanocomposites of Fe-based metallo-supramolecular polymer (polyFe) and a layered inorganic–imidazoline covalently bonded hybrid (LIIm) with different ratios (3/0.1, 0.5, 1, and 5) of polyFe/LIIm. The blue-purple-colored polyFe/LIIm composite thin films showed a pair of redox waves at 0.86–0.89 V vs. Ag/Ag^+^, which were attributed to the electrochemical redox of Fe(II/III). The appearance/disappearance of MLCT absorption was triggered by the electrochemical redox. The nanocomposites demonstrated enhanced memory properties and large optical contrast (ΔT) of 70–76% during EC changes. Among nanocomposites, the one with a ratio of 3/1 exhibited the most redshifted and strongest MLCT absorption and the longest memory. Counter anion diffusion in the composite films was lesser when combined with LIIm, while the smaller ΔE of 0.2 V showed accelerated electron transfer at the interface between the composite films and electrode. SEM images of nanocomposites suggested that LIIm nanoparticles were coated with amorphous polyFe, and the structural features of the polyFe/LIIm composites were variable with the polyFe/LIIm ratios. XRD measurement suggested that there was no intercalation of polyFe in the interlayer space between LIIm layers, possibly because of the imidazoline moiety existing between the layers and covalently bonding with the layers in LIIm. Consequently, the nano-scale composition of polyFe with LIIm was effective at improving the memory properties without a decrease in the contrast in EC changes. These results show that the nano complexing with a layered inorganic–organic covalently bonded hybrid powerfully increases the applications of metallo-supramolecular polymers as EC materials.

In addition, it was also revealed that the slight difference in the molar ratio of metallo-supramolecular polymer (MSP) and inorganic material in the nanocomposites is crucially important to enhance the electrochromic properties, probably due to different morphologies of the composites. It means that the electron and anion transfer in the nanocomposite layer during the EC changes is controllable by adjusting the molar ratio. Based on the obtained research results, we will further investigate the structural and electronic effects of MSPs (e.g., the different metal species or the different dimensions (1D, 2D, and 3D) of polymer structures) on the EC properties of nanocomposites. EC smart windows have received much attention as a new window type to reduce energy consumption of air conditioning in rooms and vehicles, and EC materials are a key to determine the performance of smart windows. The practical results obtained by this research are considered to greatly contribute to the practical application to EC smart windows using MSPs.

## Figures and Tables

**Table 1:** Retention times of the colorless state for polyFe/LIIm composite films.

	Retention Times (s) ^1^		
	Open Circuit	Closed Circuit	Average ^2^
PolyFe (reference)	236	64	150
PolyFe/LIIm-3/0.1	236	269	253
PolyFe/LIIm-3/0.5	44	53	49
PolyFe/LIIm-3/1	785	361	573
PolyFe/LIIm-3/5	557	617	587
Average ^3^	406	325	365

^1^ Retention times to recover 95%. The films spin coated on ITO glass (1.5 cm × 1.5 cm) were used for measurements. ^2^ Average values of the open and closed circuits. ^3^ Average values of the four composites.

## Data Availability

Not applicable.

## Supplementary Materials

The following are available online at https://www.mdpi.com/article/10.3390/polym14050915/s1, Figures S1–S3: SEM images of polyFe, LIIm, and polyFe/LIIm composites, Figure S4: SEM images and a histogram in particle sizes of polyFe/LIIm composite-3/1, Figure S5: CV plots.
