# Supplementary material for: Nanocomposites of Fe(II)-Based Metallo-Supramolecular Polymer and a Layered Inorganic–Organic Hybrid for Improved Electrochromic Materials
Source: Polymers (Basel). 2022 Feb 25;14(5):915. doi: 10.3390/polym14050915 (PMC8912828; doi:10.3390/polym14050915)
Supplement: Supplementary file 1 [file polymers-14-00915-s001.zip › polymers-1566860-supplementary.pdf]

Supplementary Information

Nanocomposites of Fe(II)-based metallo-supramolecular polymer and a layered inorganic–organic hybrid for improved electrochromic materials

Kazuko Fujii*, Manas Kumar Bera, Dines Chandra Santra, and Masayoshi Higuchi*

Electronic Functional Macromolecules group, National Institute for Materials Science, 1-1 Namiki, Tsukuba, Ibaraki 305-0044, Japan

* Correspondence: FUJII.Kazuko@nims.go.jp; +81-298604363 (K. F.) and HIGUCHI.Masayoshi@nims.go.jp; +81-298604744 (M. H.)

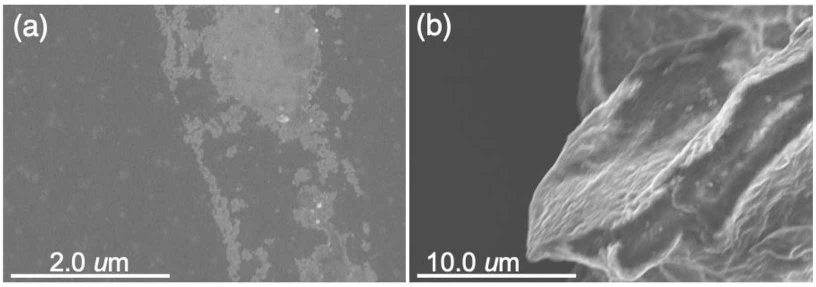

Figure S1. SEM images of polyFe.

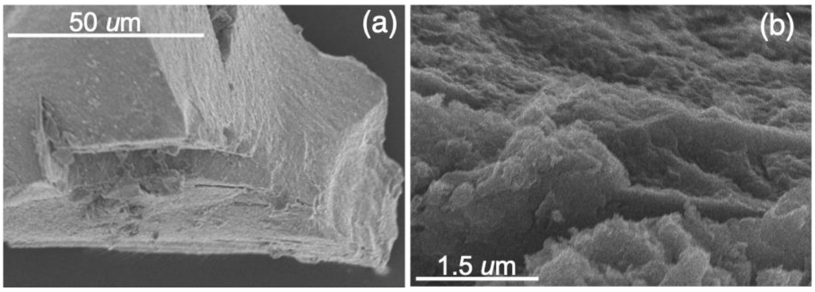

Figure S2. SEM images of layered inorganic-imidazoline covalently bonded hybrid (LIIm).

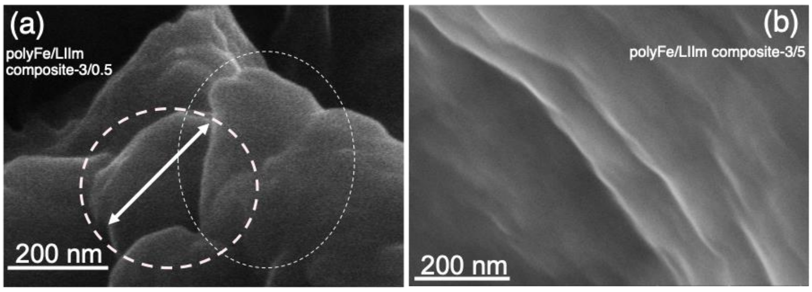

Figure S3. SEM images of polyFe/LIIm composites (a) 3/0.5 and (b) 3/5. Some particles and the size are marked with dot circles and a double arrow.

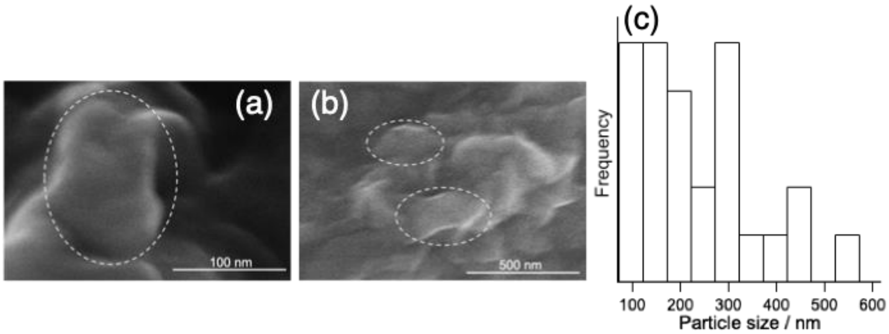

Figure S4. (a, b) SEM images of polyFe/LIIm composite-3/1 with different magnifications (some particles are marked with dot circles) and (c) a histogram in the particle size. The average particle sizes in polyFe/LIIm composites-3/0.5, -3/1 and -3/5 were determined using the observable particles, because the particles were heavily stacked and overlapped to form aggregates.

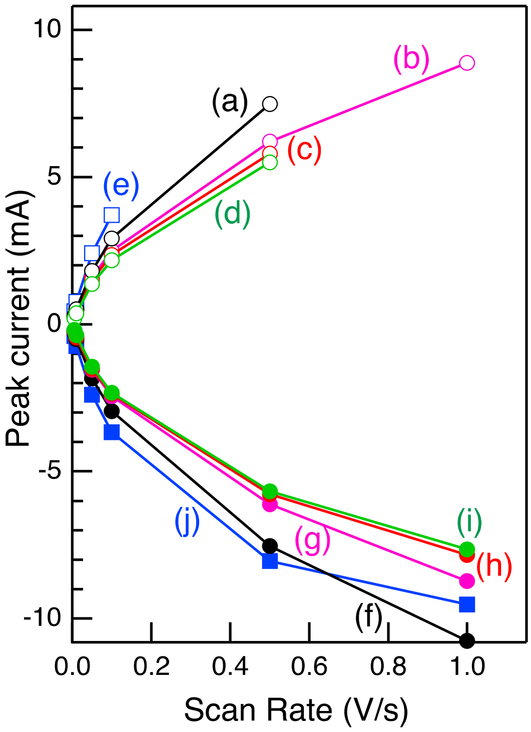

Figure S5. CV plots of the oxidation (a–e) and reduction (f–j) current as a function of the scan rates for polyFe/LIIm composites (a and f) 3/0.1, (b and g) 3/0.5, (c and h) 3/1, and (d and i) 3/5 and (e and j) reference polyFe films casted on ITO glass.
